# Scalable Upcycling of Spent Lithium‐Ion Battery Anodic Graphite to Electronic‐Grade Graphene

**DOI:** 10.1002/advs.202524344

**Published:** 2026-01-07

**Authors:** Janan Hui, Jenna N. Trost, Wesley Y. Chen, Maryam Khalaj, Lindsay E. Chaney, Peyton Melin, Albert L. Lipson, Jennifer B. Dunn, Mark C. Hersam

**Affiliations:** ^1^ Department of Materials Science and Engineering Northwestern University Evanston IL USA; ^2^ Department of Chemistry Northwestern University Evanston IL USA; ^3^ Department of Chemical and Biological Engineering Northwestern University Evanston IL USA; ^4^ Applied Materials Division Argonne National Laboratory Lemont IL USA

**Keywords:** energy storage, life cycle analysis, recycling, supercapacitors, sustainability

## Abstract

Recycling processes for lithium‐ion batteries (LIBs) are imperative to support the sustainable growth of global energy storage systems. This study introduces a scalable method for the upcycling of spent graphite anodes from LIBs to produce electronic‐grade graphene nanoplatelets. In addition to comprehensive materials characterization, the electronic quality of the upcycled graphene is demonstrated by formulating it into a screen printing ink that achieves high‐resolution patterning and thin‐film electrical conductivity exceeding 10^4^ S m^−1^. This screen printing ink is also used to print planar micro‐supercapacitors with exceptional areal capacitance (1.78 mF cm^−2^), areal energy density (0.247 µWh cm^−2^), and cycling stability (> 10 000 cycles). Life cycle assessment (LCA) and techno‐economic analysis (TEA) highlight the environmental benefits and cost reductions attainable through upcycling of graphite from LIBs. By capturing economic value from spent LIBs, this work fosters a sustainable battery supply chain and provides an abundant and geographically distributed raw material for electronic‐grade graphene.

## Graphical abstract

Graphite anodes from spent lithium‐ion batteries are upcycled into electronic‐grade graphene nanoplatelets for highly conductive screen printing inks (> 10^4 ^S m^−1^). Screen‐printed micro‐supercapacitors confirm the utility of the upcycled graphene (1.78 mF/cm^2^ capacitance for > 10 000 cycles). Life cycle assessment and techno‐economic analysis highlight significant environmental benefits and cost reductions in support of a sustainable battery supply chain.

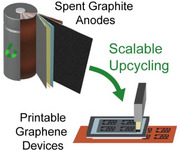

## 1 Introduction

The rapid adoption of wearable technologies, portable electronics, and electric vehicles (EVs) over the past decade has dramatically increased the production and demand for lithium‐ion batteries (LIBs). However, the finite lifespan of LIBs is leading to a corresponding surge in LIB‐related electronic waste [1, 2]. Specifically, global LIB waste is projected to rise from the estimated 464 000 tons annually in 2025 to 1.2 million tons annually in 2030, which has significant deleterious consequences especially in light of the high cost of disposal, limited waste management infrastructure, and environmental contamination from toxic heavy metals and electrolytes [3, 4, 5, 6]. Looking to the future, LIB waste production is expected to accelerate further as recent government policies worldwide are promoting investment in LIB manufacturing, creating urgency for the sustainable disposal and recycling of retired LIBs [7, 8, 9].

Current strategies for LIB recycling have significant limitations, especially from the perspective of environmental sustainability. Today, LIBs are most commonly recycled using hydrometallurgical or pyrometallurgical routes [10, 11, 12]. These methods are often non‐specific to different LIB chemistries and typically begin from black mass (i.e., a shredded mix of LIBs), leading to downstream issues in separation and purity that generate significant emissions [13, 14, 15, 16]. Moreover, these recycling efforts primarily focus on recovering and reusing metals in the cathode such as Cu, Co, Ni, and Mn [17, 18]. Although economic incentives exist to recycle these high‐value elements, it should be noted that they make up less than half of the weight of a LIB [10, 19]. Comparatively, the recycling of anodic materials, particularly graphite, has not been extensively studied despite the anode accounting for a significant portion of the weight of a LIB (∼28%) [20]. For this reason, recycled LIB anodes present a potentially substantial alternative graphite stream while simultaneously reducing electronic waste.

Recycling strategies for graphite have garnered more interest after the tightening of exports from China and its recent classification as a critical mineral by the U.S. Department of Energy, European Union, and Australia [21, 22, 23]. Yet, the development of industrial‐scale recycling systems remains stagnant due to low economic incentives and incompatibility with traditional cathode recycling methods. At the research laboratory scale, graphite recovered from spent LIBs has been directly reused for applications in catalysis, composite materials, absorbents, or reassembly into LIBs [21, 24, 25]. While these approaches contribute to a more sustainable supply chain, the compromised performance in direct LIB reuse and similar cost to graphite raw materials are barriers to creating value for recycling at the industrial scale. Therefore, a need remains for demonstrating high‐value applications for graphite recovered from LIBs, such as upcycling graphite into graphene for next‐generation electronic devices [26, 27].

The exceptional electronic conductivity, surface area, mechanical strength, and chemical stability of graphene make it an ideal candidate for electronic components, sensors, and energy storage devices [28, 29]. By 2030, it is anticipated that 10% of recycled graphite could be used to produce graphene oxide or reduced graphene oxide that achieves some properties of pristine graphene [21]. However, the widely used Hummers method for producing graphene oxide from graphite employs caustic reagents that undermine the environmental benefits of LIB graphite recycling and creates defect‐rich graphene oxide that requires a further reduction step to reach moderate electrical conductivities [30, 31]. In comparison, relatively few studies have attempted to upcycle graphite directly into pristine graphene [32, 33]. In these previous attempts, scalable processing was not employed, but instead electrochemical and ultrasonication methods were utilized with low yields and low throughput compared to other state‐of‐the‐art top‐down exfoliation techniques [34, 35, 36]. In addition, the electronic applications of these prior upcycled pristine graphene efforts have been restricted to conductive films and circuit boards fabricated through rudimentary manufacturing methods such as dip‐coating, spray deposition, or vacuum filtration [26, 32, 34] since the demonstrated electrical conductivity (< 10^4 ^S m^−1^) is insufficient for high‐value printed electronic applications [37, 38, 39]. These challenges highlight the need to develop scalable and effective processing methods that can upcycle spent LIB graphite into high‐performance graphene‐based printed electronic devices.

Herein, we employ wet jet milling (WJM), a high‐throughput liquid phase exfoliation method, to upcycle anodic graphite from spent LIBs into electronic‐grade graphene. Our upcycled electronic‐grade graphene is further formulated into printable inks compatible with screen printing, enabling roll‐to‐roll additive manufacturing of electronics and energy storage devices. As a demonstration of the utility of our upcycled graphene, planar micro‐supercapacitors (MSCs) are screen printed and shown to achieve a capacitance comparable to other graphene MSCs. Additionally, life cycle assessment (LCA) and techno‐economic analysis (TEA) were performed to quantify improvements in sustainability and economic viability of the graphite recycling process and upcycled graphene. Overall, this work paves the way for producing electronic‐grade graphene from spent LIB graphite, thereby providing an economic incentive to accelerate efforts in industrial‐scale LIB anode upcycling.

## 2 Results and Discussion

### 2.1 Scalable Upcycling of Spent LIB Anodic Graphite to Electronic‐Grade Graphene

**FIGURE 1:**
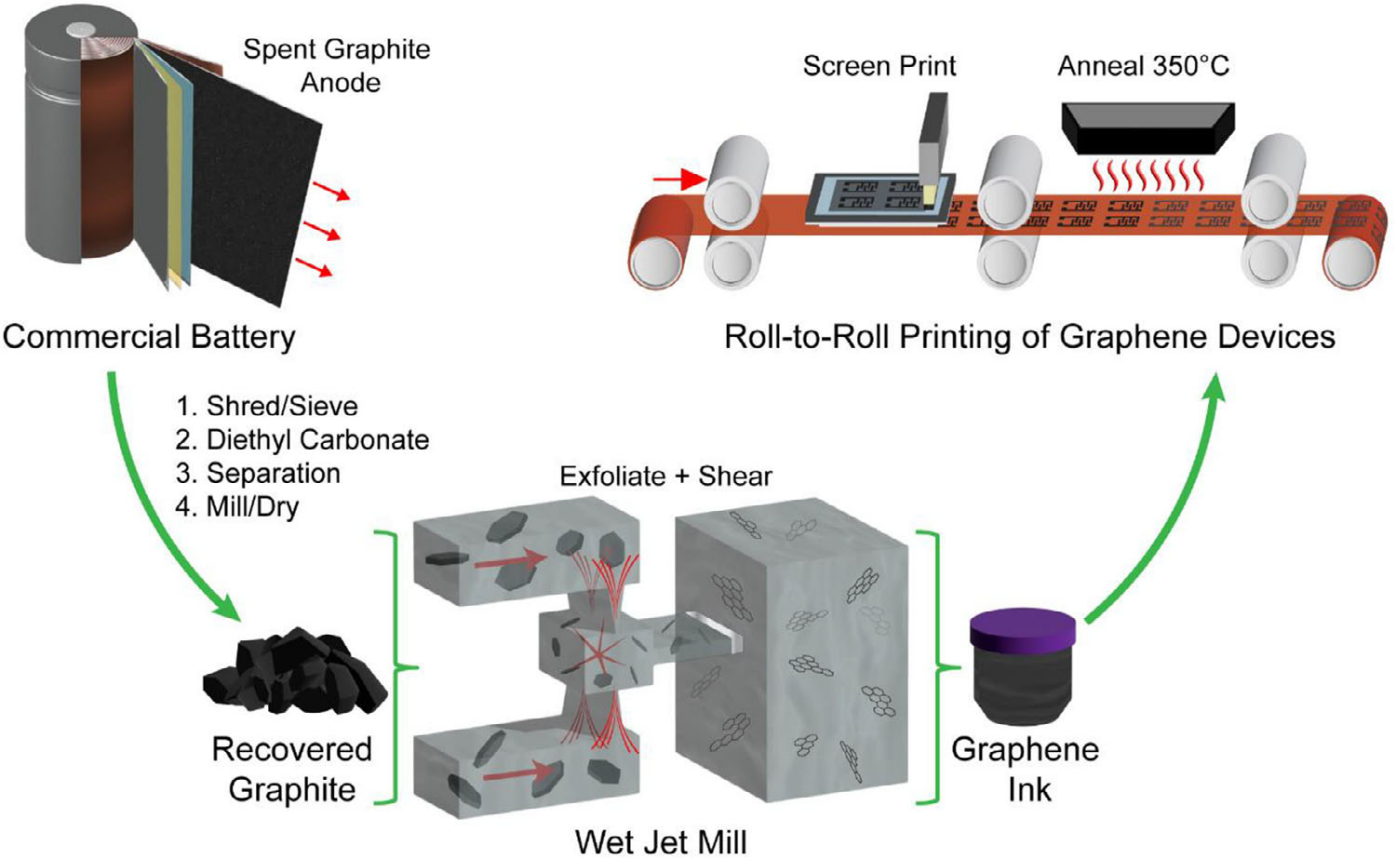
Process overview. Overview of the scalable process for upcycling spent LIB anodic graphite to electronic‐grade screen‐printable inks.

A direct recovery method with minimal processing was used to separate the anodic graphite from spent LIBs (see Experimental Section below), yielding a highly crystalline bulk graphite material to be further processed into graphene nanoplatelets (Figure 1). It is important to note that while the anode disassembly in this study was performed through a lab‐scale hand separation process, similar graphite material can be readily obtained from a previously shown commercially viable process that involves separation through shredding and mechanical separation of LIB materials [40].

**FIGURE 2:**
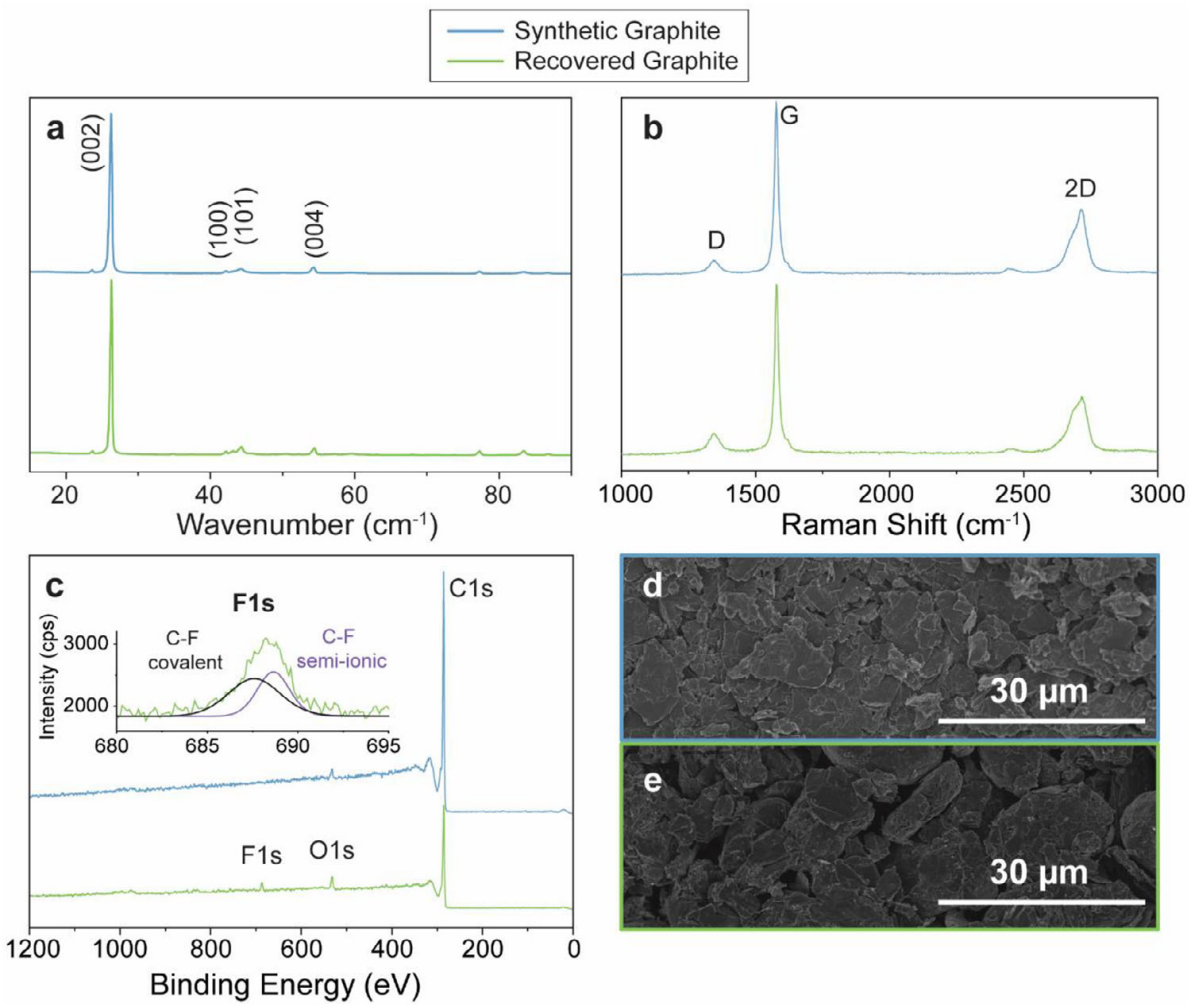
Comparison of Synthetic and Recovered Graphite. a) XRD spectra. b) Raman spectra. c) XPS survey spectra with F1s band of the recovered graphite (inset). d) SEM image of synthetic graphite powder. e) SEM image of recovered graphite powder.

X‐ray diffraction (XRD), Raman spectroscopy, X‐ray photoelectron spectroscopy (XPS), and scanning electron microscopy (SEM) were used to verify the quality of the recovered graphite prior to exfoliation (Figure 2). XRD of the recovered graphite shows a diffraction pattern with sharp peaks and intensities that closely match commercial synthetic graphite flakes (Figure 2a). Specifically, the interlayer distances (d‐spacings) as calculated by the Bragg equation differ only by 0.01 nm (0.338 and 0.337 nm, respectively), suggesting minimal variations in graphite crystal quality. Raman spectroscopy further confirms the similarity between the two types of graphite (Figure 2b). In particular, the peak intensity ratio of the *D*‐band to *G*‐band (I_D_/I_G_) for the recovered graphite was found to be 0.126 ± 0.02, which is lower than the observed value of 0.193 ± 0.03 for synthetic graphite. More importantly, the asymmetry of the 2D band with a noticeable shoulder at ∼2700 cm^−1^ is indicative of significant multilayer stacking for both graphite samples. These findings confirm that the recovered graphite is a highly crystalline material with low defect density that is suitable for graphene exfoliation. Comparison of the XPS survey spectra for both graphite types indicates minimal elemental impurities (Figure 2c), where the only observable difference for the recovered graphite is a small F1s peak at ∼688 eV (Figure 2c, inset). This small F1s peak is well fit with a combination of semi‐ionic and covalent C─F bonding, which suggests the presence of trace polyvinylidene fluoride (PVDF) remaining on the recovered anodic material. However, this residue is inconsequential as it will be removed during exfoliation and subsequent purification via centrifugation. SEM images of the recovered graphite indicate a larger particle size and more spherical morphology compared to synthetic graphite (Figure 2d,e). Overall, the recovered graphite has characteristics suitable for subsequent exfoliation to electronic‐grade graphene.

The recovered graphite was dispersed in ethanol with ethyl cellulose (EC) and subjected to liquid phase exfoliation using WJM (see Experimental Section below). The graphene yield (i.e., output graphene mass divided by input graphite mass) from this process was 4.2% after centrifugal purification, which is comparable to incumbent high‐throughput exfoliation processes [36, 41]. The concentration of the resulting graphene dispersion is high compared to previous work using spent LIB graphite (Table 1). Our graphene dispersion was then flocculated to isolate graphene‐EC powder (∼54% graphene based on thermogravimetric analysis) (Figure S1), which was then centrifugally mixed with terpineol to produce a stable screen printing ink. Screen printing of the WJM upcycled graphene produced high‐resolution features (down to ∼50 µm linewidths) with high electrical conductivity (> 10^4^ S m^−1^). This electrical conductivity exceeds the performance of prior attempts to achieve conductive graphene films based on graphite recovered from spent LIBs (Table 1).

**TABLE 1:** Comparison of conductive graphene materials upcycled from LIBs.

Exfoliation technique	Film formation	Concentration (mg mL^−1^)	Conductivity (S m^−1^)
WJM (this work)	Screen printing (this work)	2.1	36 000
Ultrasonication [26]	Vacuum filtration	0.8	9100
Hummer's method [38]	Vacuum filtration	—	543
Hummer's method [37]	Lyophilized film	—	377
Shear mixing [34]	Spin–coating	0.19	63

**FIGURE 3:**
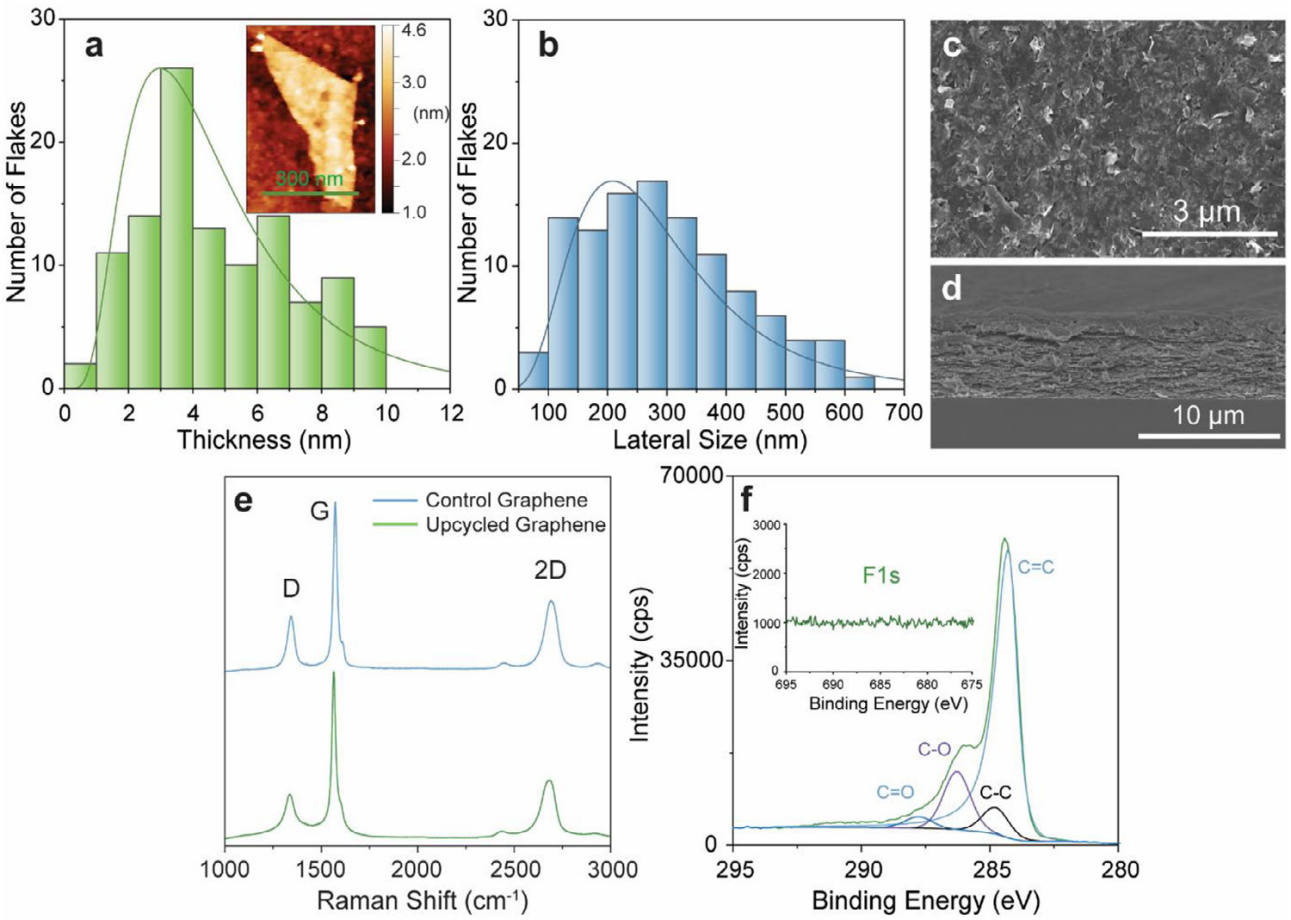
Characterization of Upcycled Graphene Nanoplatelets. a) Atomic force microscopy (AFM) thickness histogram (n = 111 nanoplatelets) fitted with a log‐normal distribution. Inset: Representative AFM image of an upcycled graphene nanoplatelet. b) AFM lateral size histogram (n = 111 nanoplatelets) fitted with a log‐normal distribution. c,d) Top‐down and cross‐sectional scanning electron microscopy images of a 5‐layer screen‐printed upcycled graphene nanoplatelet film, respectively. e) Comparison of Raman spectra between control graphene (exfoliated from synthetic graphite) and upcycled graphene. f) Fitted C1s and F1s (inset) X‐ray photoelectron spectra for the upcycled graphene powder.

Characterization of the upcycled graphene using atomic force microscopy (AFM), SEM, Raman, and XPS confirms the formation of few‐layered graphene nanoplatelets (Figure 3). The average size of the upcycled graphene nanoplatelets was found to be 293 nm laterally and 5 nm thick based on AFM images of 111 graphene nanoplatelets, indicating the successful formation of few‐layer graphene sheets (Figure 3a,b). Top‐down and cross‐sectional SEM images of screen‐printed films highlight the uniform alignment of the upcycled graphene flakes in a percolating film structure (Figure 3c,d, respectively). After annealing, the 5‐layer screen‐printed film was measured to be ∼8 µm thick through profilometry, which agrees well with the cross‐sectional SEM image. Comparison of Raman spectra between graphene exfoliated from the recovered graphite and commercial synthetic graphite flakes shows minimal differences (Figure 3e). Specifically, the I_D_/I_G_ ratio was measured to be 0.321 ± 0.03 for the upcycled graphene and 0.283 ± 0.01 for the graphene control. The I_2D_/I_G_ values can also be used to assess the quality of the graphene flakes, where the upcycled graphene showed a value of 0.407 ± 0.03 compared to the graphene control value of 0.398 ± 0.02. Importantly, XPS analysis of the upcycled graphene film confirmed the effective removal of PVDF during the exfoliation and purification procedure as evidenced by the absence of fluorine signal (Figure 3f, inset.). In addition, the C1s band was fit using an asymmetric Lorentzian line shape to reflect the high sp^2^ (C═C) content of the graphene nanoplatelets with minimal oxidation and negligible contribution from other impurities.

### 2.2 Screen‐Printed Upcycled Graphene Micro‐Supercapacitors

**FIGURE 4:**
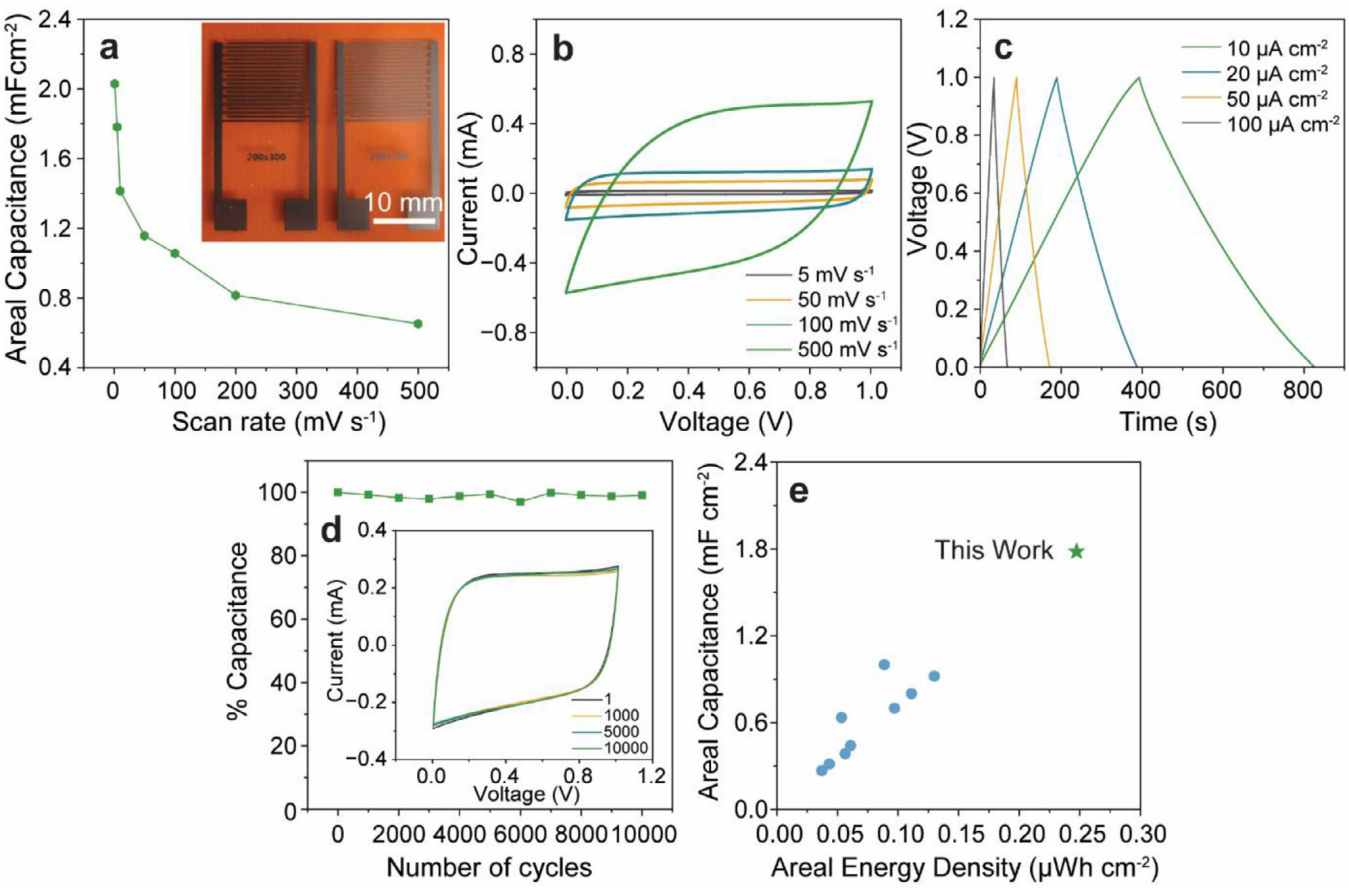
Performance of Upcycled Graphene in Printed Micro‐Supercapacitors (MSCs). a) Rate capability curve and photograph (inset) of 5‐layer screen‐printed MSCs (inset). b) Cyclic voltammetry curves at scan rates from 5 to 500 mV s^−1^. c) Galvanostatic charge–discharge curves for the screen‐printed MSCs at current densities from 10 to 100 µA cm^−2^. d) MSC capacitance retention over 10 000 cycles at a scan rate of 200 mV s^−1^, with select cyclic voltammograms from the 1st cycle to the 10 000th cycle (inset). e) Comparison of areal capacitance and energy density for recently published work on printed graphene‐only planar MSCs (please see Table S1 for a list of the references used in generating this plot).

To demonstrate the utility of the upcycled graphene nanoplatelets, planar micro‐supercapacitors (MSCs) were fabricated using screen‐printed graphene films as the electrode material (Figure 4). Specifically, a 5‐layer graphene film was patterned using a custom screen design to create interdigitated electrodes on a polyimide substrate. The electrode fingers were 200 µm wide and 10 mm long with 300 µm spacing (Figure 4a, inset). Following annealing at 350°C to remove excess EC, a PVA‐H_3_PO_4_ gel electrolyte was drop‐cast and spread onto the working area of the device followed by drying overnight. The electrochemical performance of the MSCs was assessed using cyclic voltammetry (CV) and galvanostatic charge–discharge measurements (Figure 4b–d). The near‐rectangular CV profile and triangular galvanostatic curves with no observable redox peaks indicate the excellent double‐layer capacitive behavior of the electrodes (Figure 4b,c, respectively). An areal capacitance of 1.78 mF cm^−2^ is observed at a scan rate of 5 mV s^−1^, which decreases to 652 µF cm^−2^ at a scan rate of 500 mV s^−1^ (Figure 4a). The MSCs exhibited exceptionally stable charge–discharge cycling with 98.7% capacitance retention after 10 000 cycles at a scan rate of 200 mV s^−1^ (Figure 4d). This performance rivals state‐of‐the‐art graphene MSCs, highlighting the viability of the upcycled graphene inks for high‐quality conductive electrodes [42, 43, 44]. In particular, the areal capacitance (1.78 mF cm^−2^) and areal energy density (0.247 µWh cm^−2^) achieved with these MSCs are the highest among printed graphene‐only planar MSCs in recent literature (Figure 4e). This comparison excludes alternative fabrication methods such as 3D printing, blade coating, and laser scribing, which typically lead to substantially thicker films up to millimeters in height with limitations in resolution and roll‐to‐roll compatibility (extended comparison provided in Table S1) [45]. The high performance of our upcycled graphene in MSCs demonstrates that it can be effectively included as conductive components in state‐of‐the‐art printed devices.

### 2.3 Life Cycle Assessment and Techno‐Economic Analysis

**FIGURE 5:**
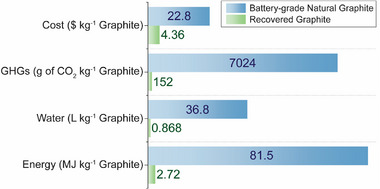
Life Cycle Assessment (LCA) and Techno‐Economic Analysis (TEA). Each metric is normalized to the following maximum values for visual clarity: 1) Cost: 100$ kg^−1^ graphite; 2) Greenhouse gas emission (GHGs): 10 000g of CO_2_ kg^−1^ graphite; 3) Water: 100 L kg^−1^ graphite; 4) Energy: 100 MJ kg^−1^ graphite.

To evaluate the advantages in recovering graphite from spent LIBs compared to battery‐grade natural graphite, life cycle assessment (LCA) was performed for greenhouse gas emissions (GHGs), water consumption, and energy consumption based on ISO 14040/44 standards (Figure 5) [46, 47]. A functional unit of 1 kg of graphite and cradle‐to‐gate system boundary were used. Graphite recovered from LIBs reduces GHGs, water, and energy by 96.7%, 97.6%, and 97.8%, respectively, compared to battery‐grade natural graphite [48]. Reductions compared to synthetic graphite (costs range between $6–10/kg; GHG emissions can reach 20 000 g CO_2_/kg) would be at least as substantial [49]. These improvements come primarily from eliminating the intensive mining and refining processes in favor of rinsing and separating steps. In addition, techno‐economic analysis (TEA) shows that the production costs of graphite recovered from LIBs are 80.9% lower compared to battery‐grade natural graphite (Figure 5). When expanding the system boundary to the production of graphene, LCA metrics (i.e., GHGs, water, and energy) for our upcycled graphene are also substantially lower than our previous work based on synthetic graphite (Table S2). Overall, the upcycling process achieves low‐cost graphene production of $5.79 per gram (Table S2), making it viable for high‐performance printed electronic applications.

Mass allocation was implemented in the LCA and TEA with treated co‐products from the spent batteries as separate waste streams (Figure S5). Direct recycling enables recovery of other battery components, reducing waste and the need for raw materials from mines. With a direct recycling approach, simultaneous recovery of all these materials can minimize waste to landfills, lowering the need for mining of these materials and bridging the gap in material availability and demand [8], which is particularly crucial for securing domestic critical material supply chains. In this manner, recycling LIB waste offers multiple transformative benefits, and upcycling spent LIB graphite into electronic‐grade graphene provides sufficiently high value to incentivize LIB anode recovery.

## 3 Conclusions

This work reports a scalable solution‐based approach to directly recover and upcycle spent graphite anodes from LIBs for electronic‐grade graphene. By utilizing this upcycled graphene in screen‐printable conductive inks, films with conductivities exceeding 10^4^ S m^−1^ were achieved. Moreover, planar MSCs were demonstrated with state‐of‐the‐art areal capacitance, energy density, and cycling stability. Evaluation of this recovery method with LCA and TEA confirmed the significant environmental benefits and cost reductions that can be achieved when graphite is recycled from spent LIBs. By pairing this anode upcycling process with direct recycling of cathode high‐value metals, strong economic incentives can be generated to encourage industrial‐scale LIB recycling. Ultimately, this work promotes a more robust battery supply chain in addition to providing a sustainable supply of graphite for high‐performance graphene‐based printed electronics.

## 4 Experimental Section

### 4.1 Materials

Anodic graphite separated from spent batteries was provided by the ReCell Center at Argonne National Laboratory. Micro‐450 graphite that was used as a control comparison was purchased from Asbury Carbons. Ethyl cellulose (EC, 4 cP), terpineol, H_3_PO_4_, and polyvinyl alcohol (MW = 31000–50000) were purchased from Sigma–Aldrich. Ethanol (200 proof) was purchased from Decon Laboratories. Polyimide films (Kapton) were purchased from DuPont.

### 4.2 Extraction of Anodic Graphite

Commercial Li‐ion battery pouch cells were disassembled in an argon‐filled glovebox by hand. The anode and cathode electrodes were separated via hand sorting, and then the anode was cut into 2 by 2 cm pieces. The anode electrode pieces were then soaked overnight in anhydrous diethyl carbonate (Sigma–Aldrich), the rinsate was decanted, and the electrodes were dried at 80°C under vacuum. The graphite was then removed from the foils by placing it in deionized water with the copper foil being sieved out. The graphite was finally vacuum‐filtered and dried at 80°C under vacuum before use in exfoliation experiments.

### 4.3 Liquid Phase Exfoliation and Ink Formulation

Recovered graphite was exfoliated based on a previously optimized procedure [36]. Briefly, ethyl cellulose (EC, Sigma–Aldrich, 4 cP) and the recovered graphite were mixed vigorously in 500 mL of ethanol (Decon Labs, 200 proof) overnight at a 3% and 10% ratio by weight, respectively. This dispersion was then directly added to a Sugino Star Burst Labo (HJP‐25005V2) wet jet mill (WJM) to shear into graphene nanoplatelets by passing through a 120 µm nozzle chamber, cycling through the WJM for a total of 30 passes (∼25 min). The exfoliated sample was then subjected to centrifugation (Beckman Coulter, Avanti J‐26 XPI) at an RCF of ≈4000 g for 30 min. The collected supernatant was then flocculated by adding a 0.04 g mL^−1^ NaCl solution and centrifuging at ≈10000 g for 7 min to crash out the graphene nanoplatelets. The resulting powder was then rinsed with deionized water using vacuum filtration and dried for storage before being formulated into upcycled graphene inks.

### 4.4 Graphite and Graphene Characterization

Graphene yield was determined from UV–vis spectroscopy (Agilent, Cary‐5000), where concentration was extracted from optical absorbance spectra according to Beer's Law at a wavelength value of 660 nm. Atomic force microscopy (Asylum Research, Cypher) was used to extract flake size statistics, where samples were drop‐casted as dilute upcycled graphene dispersions onto Si/SiO_2_ wafers followed by heating on a hot plate at 350°C for 30 min to remove excess EC. The sheet resistance of the screen‐printed samples was measured using a four‐point probe measurement system (Lucas Signatone, S‐302‐4) and a source meter (Keithley, Model 2400). The thickness of the printed films was measured using a profilometer (Veeco, Dektak 150). Raman spectroscopy (Horiba, Xplora) was performed on screen‐printed graphene films or upcycled graphene powder using a 532 nm laser with an 1800 mm grating at 10% laser power. Scanning electron microscopy (Hitachi, SU8030) was performed on the surface and cross‐sections of screen‐printed graphene films at 10 kV. X‐ray photoelectron spectroscopy (XPS) measurements were conducted using an ESCALAB 250Xi spectrometer (Thermo Fisher Scientific) with an Al Kα radiation source using a laser spot size of 500 µm. The XPS spectra were analyzed based on methods in the literature with CasaXPS software [50]. X‐ray diffraction was measured using a Smartlab Gen2 3kW, where powder samples were placed directly on a silicon zero diffraction plate. A scan width of 0.02^°^ and scan speed of 10^°^ per min were used. Thermogravimetric analysis (TGA) was measured using a Mettler Toledo TGA/DSC 3+. All TGA measurements were performed in synthetic air (8.3 × 10^−4 ^L/s) from 25 to 600°C with a ramp rate of 7.5 °C min^−1^. Confocal optical microscopy was measured using an Olympus 3D laser confocal microscope. Profilometry was measured using a Dektak profilometer with a 5.0 µm stylus radius.

### 4.5 Ink Formulation and Screen Printing of Micro‐Supercapacitors

For screen printing, the upcycled graphene/EC powder was added to terpineol at a concentration of 150 mg mL^−1^ and homogenized using a centrifugal mixer (Thinky USA, ARE‐310) with zirconia ball bearings for 40 min at 2000 rpm. The viscous ink produced was then screen printed (Hary Manufacturing Inc., 886 PC DSIV) using a customized screen design (stainless steel, 160 threads per inch mesh size) onto polyimide substrates. Specifically, upcycled graphene interdigitated micro‐supercapacitor electrodes were screen‐printed with the following dimensions: 200 µm wide fingers, 10 mm long fingers, 300 µm gaps, 20 total fingers, and overall dimensions of 10 mm × 10 mm. The printed designs were then pyrolyzed in a box furnace at 350°C for 30 min to remove the excess EC. A PVA gel electrolyte was then prepared by mixing 0.5 g of PVA (MW 31000–50000, Sigma–Aldrich) with 0.5 g of phosphoric acid (85%, Sigma–Aldrich) and 4.5 mL of deionized water. The PVA‐H_3_PO_4_ gel electrolyte was drop‐casted and spread across the fingers of the device followed by drying overnight in air. Electrochemical characterization tests, including cyclic voltammetry and galvanostatic charge–discharge tests, were performed using a potentiostat (BioLogic, VSP).

### 4.6 Life Cycle Assessment (LCA) and Techno‐Economic Analysis (TEA)

LCA and TEA were conducted to compare the extraction of graphite from spent LIBs against battery‐grade natural graphite. For LCA, a cradle‐to‐gate system boundary was implemented with a functional unit of 1 kg of graphite (Figure S5). The LCA on upcycled graphite in the manuscript used an NMC622 battery cell. The results for other battery chemistries are presented in Table S3, but minimal differences are observed as the amount of graphite per battery pack is relatively constant across different battery chemistries. Data were collected from experimental processing for graphite from spent LIBs and from Engels et al. for natural graphite [48]. Environmental burden data were obtained from the Greenhouse Gases, Regulated Emissions, and Energy Use in Transportation Model (GREET) [51]. Energy use, water consumption, and greenhouse gas emissions (GHGs) were analyzed for the two production pathways of graphite, and mass allocation was performed for graphite from spent batteries. Economic allocation was not implemented due to volatility of market prices for co‐products, such as copper or cathodes. Given the high demand for minerals, displacement is not a viable option for co‐product handling as recovery of minerals from spent batteries is not yet able to supplant demand for minerals from mines [52]. For TEA, the functional unit was one kilogram of graphite, and the system boundary was cradle‐to‐gate (Figure S5). Economic and labor data were collected from chemical and equipment suppliers, and total costs were analyzed in USD per gram of graphene for an operation lifetime of 20 years for each graphite stream. Similar to the LCA, mass allocation for the graphite was implemented from spent LIB analysis. All inventory data used are presented in Tables S4–S8.

## Conflicts of Interest

The authors declare no conflict of interest.

## Supporting information




**Supporting File**: advs73662‐sup‐0001‐SuppMat.pdf.

## Data Availability

The data that support the findings of this study are available in the supplementary material of this article.
