# Supplementary material for: Scalable Upcycling of Spent Lithium‐Ion Battery Anodic Graphite to Electronic‐Grade Graphene
Source: Adv Sci (Weinh). 2026 Jan 7;13(16):e24344. doi: 10.1002/advs.202524344 (PMC13042716; doi:10.1002/advs.202524344)
Supplement: Supplementary file 1 — Supporting File: advs73662‐sup‐0001‐SuppMat.pdf. [file ADVS-13-e24344-s001.pdf]

Supporting Information

Scalable Upcycling of Spent Lithium-Ion Battery Anodic Graphite to Electronic-Grade Graphene

*Janan Hui, Jenna Trost, Wesley Y. Chen, Maryam Khalaj, Lindsay E. Chaney, Peyton Melin, Albert L. Lipson, Jennifer B. Dunn, and Mark C. Hersam**

Recovered Graphite and Upcycled Graphene Characterization

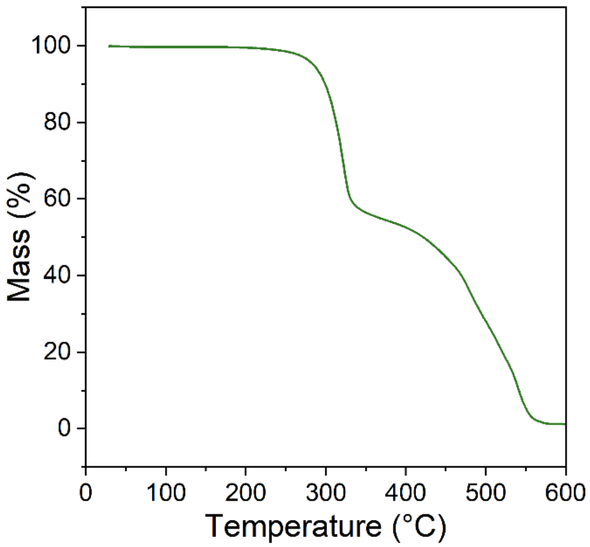

Figure S1. Thermogravimetric analysis (TGA) under open air of the graphene/EC composite produced by WJM. EC is removed at the plateau beyond ~300 °C and the reported value of ~54% graphene content is taken at 400 °C.

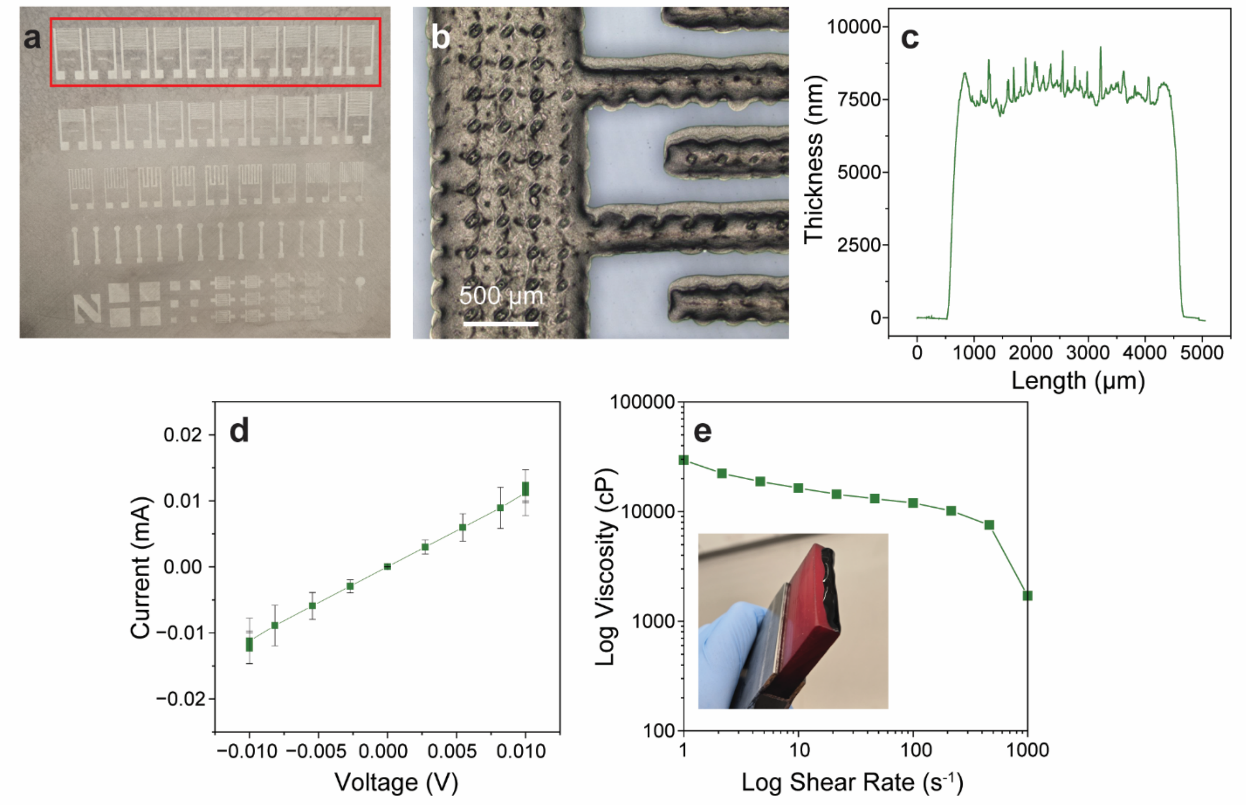

Figure S2. Screen-Printed Graphene Film Characterization. (a) Photograph of the stainless steel mesh screen used for screen printing (red box highlights the used space for design). (b) Confocal microscopy image of 5-layer screen-printed graphene electrode design on a glass microscope slide. (c) Representative profilometry scan and (d) current-voltage measurement for 5-layer screen-printed graphene on glass following annealing at 350 °C (average of 3 trials). (e) Viscosity as a function of shear rate measured for the upcycled graphene screen printing ink a photograph of the ink on a squeegee (inset).

Micro-Supercapacitor Electrochemical Characterization and Discussion

The equations and methodology to calculate areal capacitance, areal energy density, and areal power density are shown below. Areal capacitance was calculated from cyclic voltammetry (CV) data as follows:

$$C = \frac{\int_{V1}^{V2} I\, dV}{v \times \Delta V \times A} \qquad \text{(Equation S1)}$$

where the integral $\int_{V1}^{V2} I\, dV$ is found from the area under the discharge curve of the CV, ν is the scan rate, and ΔV is the scanning voltage window, yielding the capacitance value. Areal capacitance was then calculated by dividing the capacitance by the total footprint area, A, of the device including the space between the electrode fingers (100 mm^2).

Areal energy density of the device was calculated using Equation S2:

$$E = \frac{1}{2} C\Delta V \qquad \text{(Equation S2)}$$

where C is the capacitance calculated using Equation S1, and ΔV is the voltage window. To convert the units from J to Wh, E is divided by 3600 seconds. Finally, areal power density of the device was calculated using Equation S3:

$$P = \frac{E}{\Delta T} \qquad \text{(Equation S3)}$$

where E is the energy calculated using Equation S2, and Δt is the total discharge time. The electrochemical performance of the micro-supercapacitors compare favorably with other demonstrations of graphene-based devices in the literature (Table S1). Note that the extended comparison below includes examples from literature that use 3D printing, blade coating, and laser

scribing that create thicker films on a different order of magnitude than thin films from screen printing and inkjet printing. This increased thickness leads to less flexible films and also has less compatibility with roll-to-roll processes, especially in the cases of 3D printing or laser scribing. All values of energy density and power density are derived from the scan rates where the highest value of capacitance is achieved. None of the listed devices utilize recycled graphene.

Table S1. Comparison to other reported graphene planar micro-supercapacitors

Ref.	Areal Capacitance (mF cm^{-2})	Areal Energy Density (μWh cm^{-2})	Areal Power Density (μW cm^{-2})	Graphene Exfoliation Technique	Electrode Printing Technique
**This Work**	1.78	0.247	4.45	Wet jet milling	Screen
1	4900	2100	1.67	Electrochemical	3D
2	56.7	7.875	0.0011	Hummers method	3D
3	7.12	0.63	0.0142	Laser-induced	Laser
4	6.3	0.315	8.00E-04	Hummers method	DIW
5	6.16	0.5476	0.1232	Shear mixing	Blade
6	4.6	0.3	0.0045	Laser-induced	Laser
7	1	0.0888	0.002	Commercial source	Screen
8	0.92	0.13	0.0023	Wet jet milling	Screen
9	0.8	0.1111	4.00E-04	Electrochemical	Spray
10	0.7	0.09722	0.0044	Hummers method	IJP
11	0.635	0.0535	0.0025	Sand milling	Screen
12	0.441	0.061	4.00E-04	Electrochemical	IJP
13	0.385	0.0564	0.00193	Wet jet milling	Screen
14	0.313	0.0434	8.00E-04	Electrochemical	IJP
15	0.268	0.0372	0.0013	Shear mixing	IJP

*IJP = inkjet printing; DIW = direct ink writing

Life Cycle Assessment (LCA) and Techno-Economic Analysis (TEA)

Table S2. Cradle-to-gate LCA and TEA results for upcycled graphene compared to previous work with WJM graphene

	**Cost (\$/g graphene)**	**GHGs (gCO_2e/g graphene)**	**Water (L/g graphene)**	**Energy (MJ/g graphene)**
This Work*	5.79	675	15	24
Chaney et al.[8]	-	1763	20	30

*For nickel manganese cobalt 622 (NMC622) battery chemistry

Table S3. Cradle-to-gate LCA and TEA results for upcycled graphene for different battery chemistries

**Battery Chemistry**	**Cost (\$/g graphene)**	**GHGs (gCO2e/g graphene)**	**Water (L/g graphene)**	**Energy (MJ/g graphene)**
Lithium iron phosphate (LFP)	5.78	675.3	15.11	24.51
Lithium manganese oxide (LMO)	5.78	675.1	15.10	24.50
Nickel cobalt aluminum (NCA)	5.79	675.6	15.11	24.51
NMC111	5.79	675.4	15.11	24.51
NMC532	5.79	675.4	15.11	24.51
NMC622	5.79	675.5	15.11	24.51
NMC811	5.79	675.7	15.11	24.51

*NMC: nickel manganese cobalt

Minimal differences are observed between the environmental burdens and economic costs of extracting graphite from spent battery cells of different chemistries, which is likely because the amount of graphite in each cell is relatively constant (ranging from 0.156 kg to 0.207 kg graphite per kg of spent battery) across battery chemistries.

Table S4. Environmental life cycle inventory data per input kilogram

Process	Input	Energy Consumption (MJ/kg input)	Water Consumption (L/kg input)	GHG Emissions (gCO_2e/kg input)
Graphite Extraction	Diethyl carbonate[16]	10.0	1.2	373.0
Graphene Production	Ethanol[16]	51.0	39.9	359.0
	Ethyl cellulose[8]	11.1	25.0	330.0
	Salt water (1.74:1)[16]	0.13	1.0	9.2

Table S5. Environmental life cycle inventory data by processing step

Process	Step	Input	Amount	Unit
Graphite Extraction[16]	Grinding	Electricity	0.34	MJ/kg input
	Cutting	Electricity	0.34	MJ/kg input
	Rinsing	Diethyl carbonate	0.01	kg/kg input
	Drying	Electricity	4.16	MJ/kg input
	Sieving	Electricity	0.09	MJ/kg input
Graphene Production[8]	Wet Jet Milling	Ethyl cellulose	0.47	kg
		Graphite powder	1.58	kg
		Ethanol	20	L
		Electricity	4.0	MJ/kg graphite

	Centrifuge	Electricity	19	MJ/kg input
	Flocculation	Salt water	1.74	kg/kg input
		Electricity	2.62	MJ/kg input
	Dryer & Vacuum	Electricity	4.16	MJ/kg input

Table S6. Environmental life cycle inventory data by processing step

Process	Step	Total Energy Consumption (MJ)	Total Water Consumption (L)	Total GHG Emissions (gCO_2e)
Graphite Extraction	Grinding	232.3	74.7	13,055
	Cutting	67.8	21.8	3,812
	Rinsing	9.7	1.2	364
	Drying	629.0	202.2	35,349
	Sieving	14	4.5	785
Graphene Production	Wet Jet Milling	823.2	646.3	6537
	Centrifuge	645.2	207.4	36,261
	Flocculation	94.4	61.4	5361
	Dryer & Vacuum	393.3	126.4	22,102

Table S7. Techno-economic data for operating costs

Input	Per Unit	Cost (USD$/unit)*
Centrifuge bottles	bottle	169
Diethyl carbonate	kg	1499.50
Electricity (City of Chicago)	kWh	0.16
Ethanol	kg	4.64
Ethyl cellulose	kg	998
Labor	hour	21.83
Graphite	kg	22.88
Sodium chloride (for salt water)	kg	3.42
Water (City of Chicago)	ft^3	0.03

*Values from manufacturer quotes

Table S8. Techno-economic data for capital costs

Item	Cost (USD$/machine)*	Operation Years
Avanti J-26 XPI centrifuge	49,749	10
JLA 8.1000 rotor	21,630	7
Sugino Starburst Labo	67,860	10
Thermal Care Chiller	6,841	10

*Values from manufacturer quotes

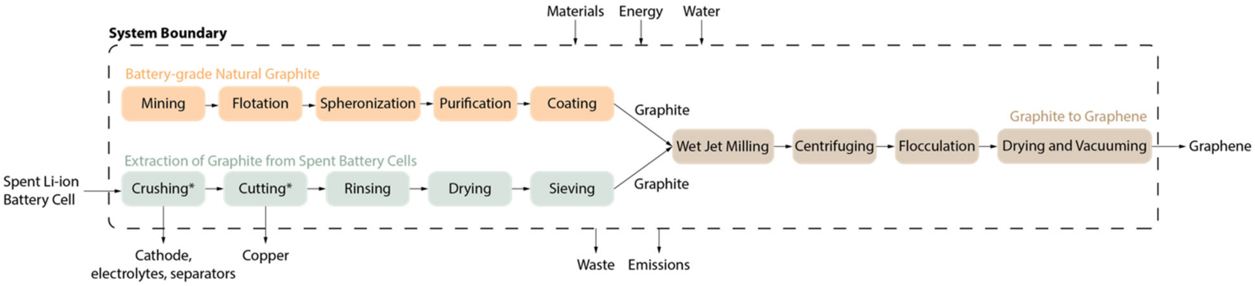

Figure S5. System boundary diagram for LCA and TEA. *These steps are handled with mass allocation.

References

(1) Zhang, L.; Qin, J.; Das, P.; Wang, S.; Bai, T.; Zhou, F.; Wu, M.; Wu, Z. Electrochemically Exfoliated Graphene Additive-Free Inks for 3D Printing Customizable Monolithic Integrated Micro-Supercapacitors on a Large Scale. *Adv. Mater.* **2024**, *36*, 2313930.

(2) Li, W.; Li, Y.; Su, M.; An, B.; Liu, J.; Su, D.; Li, L.; Li, F.; Song, Y. Printing Assembly and Structural Regulation of Graphene towards Three-Dimensional Flexible Micro-Supercapacitors. *J. Mater. Chem. A* **2017**, *5*, 16281–16288.

(3) Liu, T.; Ren, R.; Qi, Z.; Hu, J.; Chen, Y.; Huang, Y.; Guo, Y.; Cao, H.; Liang, M.; Sun, J.; Wei, J.; Zhang, H.; Zhang, X.; Wang, H. High-Performance Micro Supercapacitor Assembled by Laser-Induced Graphene Electrode and Hydrogel Electrolyte with Excellent Interfacial Wettability for High Capacitance. *J. Power Sources* **2024**, *602*, 234307.

(4) Shen, D.; Zou, G.; Liu, L.; Zhao, W.; Wu, A.; Duley, W. W.; Zhou, Y. N. Scalable High-Performance Ultraminiature Graphene Micro-Supercapacitors by a Hybrid Technique Combining Direct Writing and Controllable Microdroplet Transfer. *ACS Appl. Mater. Interfaces* **2018**, *10*, 5404–5412.

(5) Nemala, S. S.; Fernandes, J.; Rodrigues, J.; Lopes, V.; Pinto, R. M. R.; Vinayakumar, K. B.; Placidi, E.; De Bellis, G.; Alpuim, P.; Sampaio, R. S.; Montemor, M. F.; Capasso, A. Sustainable Graphene Production for Solution-Processed Microsupercapacitors and Multipurpose Flexible Electronics. *Nano Energy* **2024**, *127*, 109781.

(6) Coelho, J.; Correia, R. F.; Silvestre, S.; Pinheiro, T.; Marques, A. C.; Correia, M. R. P.; Pinto, J. V.; Fortunato, E.; Martins, R. Paper-Based Laser-Induced Graphene for Sustainable and Flexible Microsupercapacitor Applications. *Microchim. Acta* **2023**, *190*, 40. https://doi.org/10.1007/s00604-022-05610-0.

(7) Shi, X.; Pei, S.; Zhou, F.; Ren, W.; Cheng, H.-M.; Wu, Z.-S.; Bao, X. Ultrahigh-Voltage Integrated Micro-Supercapacitors with Designable Shapes and Superior Flexibility. *Energy Environ. Sci.* **2019**, *12*, 1534–1541.

(8) Chaney, L. E.; Van Beek, A.; Downing, J. R.; Zhang, J.; Zhang, H.; Hui, J.; Sorensen, E. A.; Khalaj, M.; Dunn, J. B.; Chen, W.; Hersam, M. C. Bayesian Optimization of Environmentally Sustainable Graphene Inks Produced by Wet Jet Milling. *Small* **2024**, *20*, 2309579.

(9) Liu, Z.; Wu, Z.; Yang, S.; Dong, R.; Feng, X.; Müllen, K. Ultraflexible In-Plane Micro-Supercapacitors by Direct Printing of Solution-Processable Electrochemically Exfoliated Graphene. *Adv. Mater.* **2016**, *28* (11), 2217–2222.
(10) Li, J.; Sollami Delekta, S.; Zhang, P.; Yang, S.; Lohe, M. R.; Zhuang, X.; Feng, X.; Östling, M. Scalable Fabrication and Integration of Graphene Microsupercapacitors through Full Inkjet Printing. *ACS Nano* **2017**, *11*, 8249–8256.
(11) Chen, H.; Zhang, Y.; Ma, Y.; Chen, S.; Wu, Y.; Lu, Y.; Ren, H.; Xin, S.; Bai, Y. Sand-Milling Exfoliation of Structure Controllable Graphene for Formulation of Highly Conductive and Multifunctional Graphene Inks. *Adv. Mater. Interfaces* **2021**, *8*, 2000888.
(12) Sollami Delekta, S.; Östling, M.; Li, J. Wet Transfer of Inkjet Printed Graphene for Microsupercapacitors on Arbitrary Substrates. *ACS Appl. Energy Mater.* **2019**, *2*, 158–163.
(13) Bellani, S.; Petroni, E.; Del Rio Castillo, A. E.; Curreli, N.; Martín-García, B.; Oropesa-Nuñez, R.; Prato, M.; Bonaccorso, F. Scalable Production of Graphene Inks via Wet-Jet Milling Exfoliation for Screen-Printed Micro-Supercapacitors. *Adv. Funct. Mater.* **2019**, *29*, 1807659.
(14) Sollami Delekta, S.; Adolfsson, K. H.; Benyahia Erdal, N.; Hakkarainen, M.; Östling, M.; Li, J. Fully Inkjet Printed Ultrathin Microsupercapacitors Based on Graphene Electrodes and a Nano-Graphene Oxide Electrolyte. *Nanoscale* **2019**, *11*, 10172–10177.
(15) Hyun, W. J.; Secor, E. B.; Kim, C.; Hersam, M. C.; Francis, L. F.; Frisbie, C. D. Scalable, Self-Aligned Printing of Flexible Graphene Micro-Supercapacitors. *Adv. Energy Mater.* **2017**, *7*, 1700285.
(16) Dai, Q.; Spangenberger, J.; Ahmed, S.; Gaines, L.; Kelly, J.; Wang, M. EverBatt: A Closed-Loop Battery Recycling Cost and Environmental Impacts Model; ANL--19/16, 1530874, 153050; **2019**; p ANL--19/16, 1530874, 153050.
